# Highly pathogenic avian influenza (HPAI) H5 virus exposure in domestic cats and rural stray cats, the Netherlands, October 2020 to June 2023

**DOI:** 10.2807/1560-7917.ES.2024.29.44.2400326

**Published:** 2024-10-31

**Authors:** Mirjam B H M Duijvestijn, Nancy N M P Schuurman, Johannes C M Vernooij, Michelle A J M van Leeuwen, Judith M A van den Brand, Jaap A Wagenaar, Frank J M van Kuppeveld, Herman F Egberink, Cornelis A M de Haan, Josanne H Verhagen

**Affiliations:** 1 Clinical Infectiology, Division of Infectious Diseases and Immunology, Department of Biomolecular Health Sciences, Faculty of Veterinary Medicine, Utrecht University, Utrecht, The Netherlands; 2 Section of Virology, Division of Infectious Diseases and Immunology, Department of Biomolecular Health Sciences, Faculty of Veterinary Medicine, Utrecht University, Utrecht, The Netherlands; 3 Section Farm Animal Health, Department of Population Health Sciences, Faculty of Veterinary Medicine, Utrecht University, Utrecht, The Netherlands; 4 Stray Cat Foundation Netherlands, Nieuw-Beijerland, The Netherlands; 5 Division of Pathology, Department of Biomolecular Health Sciences, Faculty of Veterinary Medicine, Utrecht University, Utrecht, The Netherlands

**Keywords:** Serosurveillance, zoonosis, pandemic preparedness, avian influenza

## Abstract

**Background:**

Highly pathogenic avian influenza (HPAI) H5Nx and human H1N1pdm2009 influenza viruses can infect cats. Infections in cats may result in viral adaptations or recombinant viruses, which may facilitate zoonotic transfer.

**Aim:**

We aimed to investigate the presence of HPAI H5 clade 2.3.4.4 and H1 influenza viruses and antibodies to these viruses in domestic and rural stray cats in the Netherlands and factors associated with exposure.

**Methods:**

Sera from stray and domestic cats, sampled 2020–2023, were analysed by ELISA and confirmed by hemagglutination inhibition assay (HAI) and pharyngeal swabs and lung tissue for influenza A virus by RT-qPCR.

**Results:**

In 701 stray cats, 83 (11.8%; 95% confidence interval (CI): 9.5–14.5) sera were positive for HPAI H5 and 65 findings were confirmed. In HAI, two sera were positive for both HPAI H5 and H1. In 871 domestic cats, four (0.46%; 95% CI: 0.13–1.2) sera were HPAI H5 positive and none were confirmed but 40 (4.6%; 95% CI: 3.3–6.2) sera were seropositive for H1 and 26 were confirmed. Stray cats living in nature reserves (odds ratio (OR) = 5.4; 95% CI: 1.5–20.1) and older cats (OR = 3.8; 95% CI: 2.7–7.1) were more likely to be HPAI H5 seropositive. No influenza A virus was detected in 230 cats.

**Conclusion:**

The higher HPAI H5 seroprevalence in stray cats compared with domestic cats suggests more frequent viral exposure, most likely due to foraging on wild birds. In contrast, exposure to H1 was more common in domestic cats compared with stray cats.

**Key public health message**

**What did you want to address in this study and why?**

Highly pathogenic avian influenza (HPAI) viruses have spread among poultry and wild birds. Avian and human influenza viruses can infect cats, and cats may function as a source of novel influenza A viruses to humans. We analysed serum samples, throat and lung samples from domestic and stray cats to investigate exposure to influenza viruses and associated factors.

**What have we learnt from this study?**

Of the 701 stray cats sampled, 83 had been exposed to HPAI virus, whereas only four of the 871 domestic cats. Exposure was more common in older cats and cats living in nature reserves. Some stray cats had been exposed to both avian and human influenza viruses. In contrast, 40 domestic cats were exposed to human influenza viruses.

**What are the implications of your findings for public health?**

We showed that cats in the Netherlands were exposed to human and/or avian influenza viruses. We recommend close monitoring of infections with influenza viruses in cats and protective measures when handling suspected cats. Further studies are needed to understand how cats become infected and whether cats can transmit highly pathogenic avian influenza virus to other animals or humans.

## Introduction

Highly pathogenic avian influenza (HPAI) H5Nx viruses of the 2.3.4.4b lineage have been enzootic in both wild and domestic birds worldwide since 2020 [1,2]. These viruses have reassorted with low pathogenic avian influenza (LPAI) H5 viruses, resulting in several neuraminidase (NA) subtype combinations [3]. Though HPAI H5Nx viruses primarily infect birds, infections are increasingly reported in carnivorous mammals, and recently also in cattle in the United States (US) [4-9]. Moreover, HPAI H5Nx infection and clinical disease have been reported in cats [10-12]. Mostly, these were sporadic cases, although three outbreaks among cats in Poland (46 cats), South Korea (40 cats) and the US (12 cats) have been reported [8,13-15]. In cats, infection with HPAI H5Nx is associated with respiratory or neurologic clinical signs and mortality, similar signs as described in other mammals [6,9,10,12-17]. However, HPAI H5Nx virus and antibodies to HPAI H5 are also found in clinically healthy cats [11,18,19]. Cat-to-cat transmission of HPAI H5Nx virus has been demonstrated in experimental settings [20,21]. Furthermore, cat-to-cat transmission of influenza A viruses (IAV) under field conditions has been described for avian origin H3N2 and H7N2 IAV and for human origin H1N1pdm2009 influenza A virus [22-25]. Although H1N1pdm2009 cat-to-cat-transmission was confirmed in experimental settings, human-to-cat-transmission, causing mild to moderate clinical signs, is considered more common [24-26].

Given that cats are susceptible to both human and avian IAV, simultaneous infection with these viruses may occur [20-24]. Concurrent infection could generate IAV recombinants with an enhanced zoonotic potential [20,21,24]. Moreover, replication of HPAI H5Nx in mammalian hosts may induce viral adaptations that facilitate replication and spread in mammalian hosts including humans. In view of the potential zoonotic risk involved with IAV infections, it is important to know the HPAI H5Nx and/or H1N1pdm2009 presence or seroprevalence in cats as an indicator of prior exposure.

Here, exposure to HPAI H5Nx and H1N1pdm2009 was investigated in domestic cats and rurally living stray cats, sampled in the Netherlands between 2020 and 2023. In addition, we analysed factors potentially associated with exposure to these viruses.

## Methods

### Sample collection

#### Blood samples

Surplus sera, used for feline immunodeficiency virus (FIV) antibody or feline leukaemia virus (FeLV) antigen detection, were collected from 701 stray cats (referred to as Stray) during trap-neuter-return-and-care activities at 72 sampling sites in 10 of 12 provinces in the Netherlands between October 2020 and June 2023 [27]. Cats with owner, based on the presence of an identification chip, were excluded from sampling. Selective sampling of apparently healthy cats excluded severely diseased or dead cats [27]. In 2023, stray cat sampling was targeted towards regions where stray cat samples were positive in ELISA for HPAI H5 antibodies, 2020–2022. Surplus samples from 871 domestic cats were collected between November 2020 and March 2023 and categorised based on sampling period and sample type into three domestic cat cohorts (referred to as Domestic). Domestic 1 and Domestic 3 consisted of surplus sera from a diagnostic laboratory, Domestic 2 of blood clots collected from the cardiac lumen from cats sent in for necropsy.

Blood clots were diluted with 200 µL phosphate buffered saline (Lonza, Walkersville, US), vortexed, centrifuged, and the supernatant was used in the assays. To confirm that these could be used as a proxy for serum, the samples were analysed for feline coronavirus (FCoV)-binding antibodies, and 31.7% (66/208) were seropositive, as expected for domestic cats [27]. However, the mean FCoV optical density (OD) value of 0.82 (standard deviation (SD): 0.32; range: 1.11) in the samples in Domestic 2 was substantially lower than the mean FCoV OD value (2.7; SD: 0.96; range: 3.6) in a subset of 407 stray cat sera with a similar seroprevalence (33.7%) [27]. Thus, we concluded that antibody detection is possible in diluted blood clots, however, low levels of antibodies may remain undetected.

#### Pharyngeal swabs or tissue samples

From 16 stray cats with respiratory clinical signs (Stray, year 2022), besides serum, pharyngeal swabs were collected in RNA shield medium (Zymo Research, Irvine, US) and stored at − 80°C. In addition, from six deceased stray cats (year 2022) with severe respiratory and/or neurologic clinical signs, pharyngeal swabs and lung tissue were collected during necropsy.

From 208 domestic cats sent in for necropsy (Domestic 2, 2021–2022), pharyngeal swabs and lung tissues were collected and stored at − 80°C. These cats were submitted for diagnostic purposes not related to a clinical suspicion of IAV infection.

#### Sample metadata

From stray cats, data on estimated age, sex, FIV/FeLV status, sampling site (municipality or postal code) and type of site (i.e. dairy farms, industrial areas, countryside residences, holiday parks or camp sites and nature reserves) and sampling year were available [27].

From domestic cats, data on age, sex, sampling site (postal code) and sampling year were available. Data regarding FIV/FeLV status, type of location and outdoor access were unavailable.

### Laboratory methods

#### Detection of antibodies to avian influenza virus

##### ELISA

Binding antibodies to the haemagglutinin (HA) protein of HPAI H5N8 clade 2.3.4.4c [28] (HA of A/Chicken/NL/14015526/2014, carrying a single substitution, R227S, referred to as HPAI H5c) and IAV H1N1pdm2009 (HA of A/California/04/2009, carrying T200A and E227A substitutions, referred to as H1) in cat sera were detected using an ELISA, as described previously [19], using 1:100 diluted sera. The mutant H1 protein was selected based on its increased hemagglutinating ability [19,29].

Subsequently, to explore cross-reactivity in the cat sera, the presence of binding antibodies to the recombinant HA proteins of a more recent [28] HPAI H5N1 clade 2.3.4.4b virus (A/Common Tern/NL/26/2022) (HPAI H5b) and a LPAI H5N2 virus (A/Common Teal/NL/4/2022) (LPAI H5) was investigated in serum samples that tested positive for HPAI H5c and/or H1.

The ELISA detects binding antibodies to multiple epitopes on the HA1 and HA2 domain of the HA protein [19]. The HA amino acid sequence identity of the H5c and H5b proteins, as shown in Supplementary Figure S1E, was 96.6%, and the location of the 11 amino acid differences between these two proteins was not associated with antigenic changes as determined in a hemagglutination inhibition assay (HAI) [30].

A detailed description of IAV subtypes used for the production of HA proteins or reference antisera, including a sequence identity matrix and phylogenetic tree, is available in Supplementary Table S1.

##### Hemagglutination inhibition assay (HAI)

The HAI is considered the gold standard in IAV antibody detection as it detects receptor-binding epitopes of IAVs. It has a higher specificity, but lower sensitivity than the ELISA [19]. The presence of receptor-binding antibodies for HPAI H5c and/or H1 virus in ELISA-positive sera was investigated with HAI. We used 55 negative sera as additional negative controls. The same HPAI H5c (A/Chicken/NL/14015526/2014) and H1 (A/California/04/2009) virus HA proteins were used in HAI as were used in the ELISA but conjugated to SpyCatcher-mi3 nanoparticles [19]. Samples were analysed according to Zhao et al. 2020 [19] with the following adaptations: twofold serial dilutions starting at 1:20 and chicken red blood cells (RBCs) 0.5% were used in the assays. To remove non-specific agglutinins, the sera were pre-incubated with 50% packed RBCs at 4°C for 1 hour. To detect residual non-specific serum hemagglutination activity, a serum control without HA-mi3 nanoparticles was performed. The cut-off was set at 1:40, and sera with a residual non-specific serum hemagglutination activity > 1:40 were removed and marked invalid. The antibody titre was defined as the highest dilution of the serum showing complete inhibition of hemagglutination using 4–8 hemagglutinating units per 25 µL. Additionally, cat sera with a sufficient volume that were ELISA positive for HPAI H5c or H1 and the subset of negative sera, were analysed in a HAI using LPAI H5N3 (A/Mallard/Netherlands/96/2019, referred to as LPAI H5N3).

##### Description negative and positive control sera

Supplementary Figure S1F and Supplementary Table S1 show the sheep and ferret polyclonal reference antisera used as positive controls. The HA amino acid sequence identity between the reference sera and their homologous strains is shown in Supplementary Figures S1E and F and was at least 98%. A specific pathogen-free (SPF) cat serum was used as a negative control to determine the ELISA cut-off. Cat sera were considered positive when their ELISA OD value was ≥ 5 times the OD value of the SPF cat serum [19]. The results were normalised per ELISA plate and expressed as ratios (ELISA OD value divided by the cut-off). A subset (n = 55) of stray cat sera that tested ELISA negative for HPAI H5c and H1 was used as an additional negative control cohort in the ELISA HPAI H5b, ELISA LPAI H5, HAI HPAI H5c, HAI LPAI H5 and HAI H1 assays.

#### Detection of influenza A virus (IAV)

In the reverse transcriptase-quantitative PCR (RT-qPCR) analysis, we used a tiered approach to maximise efficiency and minimise costs, where low-at-risk samples were pooled but high-at-risk samples were not. From stray cats, pharyngeal swabs and lung tissue were analysed individually. From domestic cats, 41 of 208 autopsy reports noted severe respiratory and/or neurologic abnormalities. For virus detection in these cats, pharynx swab and lung tissue were pooled per cat. From 167 of 208 cats, pharynx swabs and lung samples were pooled per three cats.

To detect IAV RNA, a generic RT-qPCR targeting the IAV matrix gene [31] was optimised for detection of more recent IAV strains. The primers and probes used in the RT-qPCR are described in Supplementary Table S2. A quantification cycle (Cq) value > 39 was considered negative. Glyceraldehyde-3-phosphate dehydrogenase (GAPDH) was used as an internal control, and as an internal extraction control, phocine distemper virus (PDV) was added to the sample before RNA extraction, see Supplementary Table S2 for more details.

### Data analysis

Data were analysed using SPSS (IBM SPSS Statistics for Windows, version 28.0, IBM Corp., Armonk, US) and GraphPad (GraphPadPrism version 10 for Windows, https://www.graphpad.com). The sample sites of stray cats were mapped using Datawrapper (https://www.datawrapper.de). To calculate the occurrences, the exact binominal test was used. The mean rank of ELISA OD ratios was compared using the non-parametric Kruskal-Wallis test with Dunn’s correction for multiple comparisons. We performed a univariable risk analysis on putative factors associated with ELISA HPAI H5c seropositivity using the χ2 or Fisher’s exact test (frequency ≤ 5). Factors included for stray cats were estimated age, sex, FIV status, type of sampling site and sampling year and for domestic cats age, sex and sampling year. None of the 698 stray cat sera used in our study tested positive for FeLV (0%; 95% confidence Interval (CI): 0.0–0.5%) therefore this factor was not included in the analysis. In addition, for stray cats, a multivariable binary logistic regression was performed to assess the strength of association of these factors with ELISA HPAI H5c seropositivity. The results of the full model were presented as frequency tables with odds ratio (OR) with 95% CI. The estimated stray cat age was categorised into age < 3 years and age ≥ 3 years as described earlier. This breakpoint ensured that each category contained at least 100 cats [27]. The statistical significance was set to p < 0.05 [27].

## Results

### Sample collection and description

**Figure 1:**
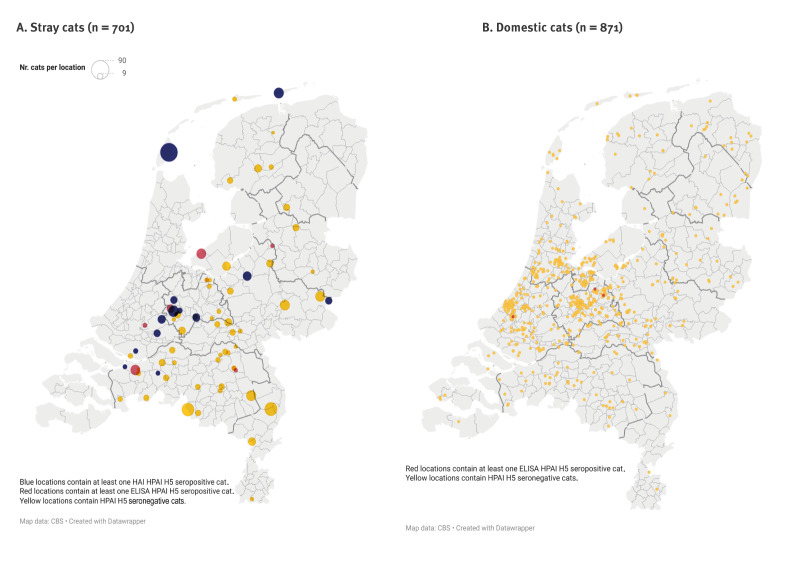
Spatial distribution of stray (n = 701) and domestic (n = 871) cats serologically tested for highly pathogenic avian influenza (HPAI) H5 antibodies, the Netherlands, October 2020–June 2023^a^

ELISA: enzyme linked immunosorbent assay; H: hemagglutinin; HAI: hemagglutination inhibition assay; HPAI: highly pathogenic avian influenza.

^a^ Information on spatial location was available for 758 domestic cats.

The cats (n = 1,572) were sampled at various locations within the Netherlands (Figure 1A,B). While stray cat samples originated from rural areas, domestic cat samples predominantly originated from more urbanised areas. Most (548/701; 78.2%) stray cats were estimated [27] aged < 3 years while most domestic cats (657/834; 78.8%) were aged ≥ 3 years. Information on age was unavailable for 37 domestic cats. The male to female ratio for the stray cats was 0.82 (314:385) and 1.4 (485:352) for the domestic cats. Information on sex was missing from two stray and 34 domestic cats.

In the HPAI H5c ELISA, the HA protein of A/Chicken/NL/14015526/2014 was used.

Panel A. The diameter of the circle represents the number of cats sampled per 72 sampling sites. The smallest circle depicts one cat, the largest sample depicts 85 cats (mean: 25.2; median: 16.0; interquartile range (IQR): 8.0–27.0).

Panel B. The circles represent individual domestic cat serum samples.

The sites were plotted using coordinates extracted from the postal code. When the postal code was unavailable, the coordinates of the municipality centre were used.

### Antibody detection

#### Stray cats

**Figure 2:**
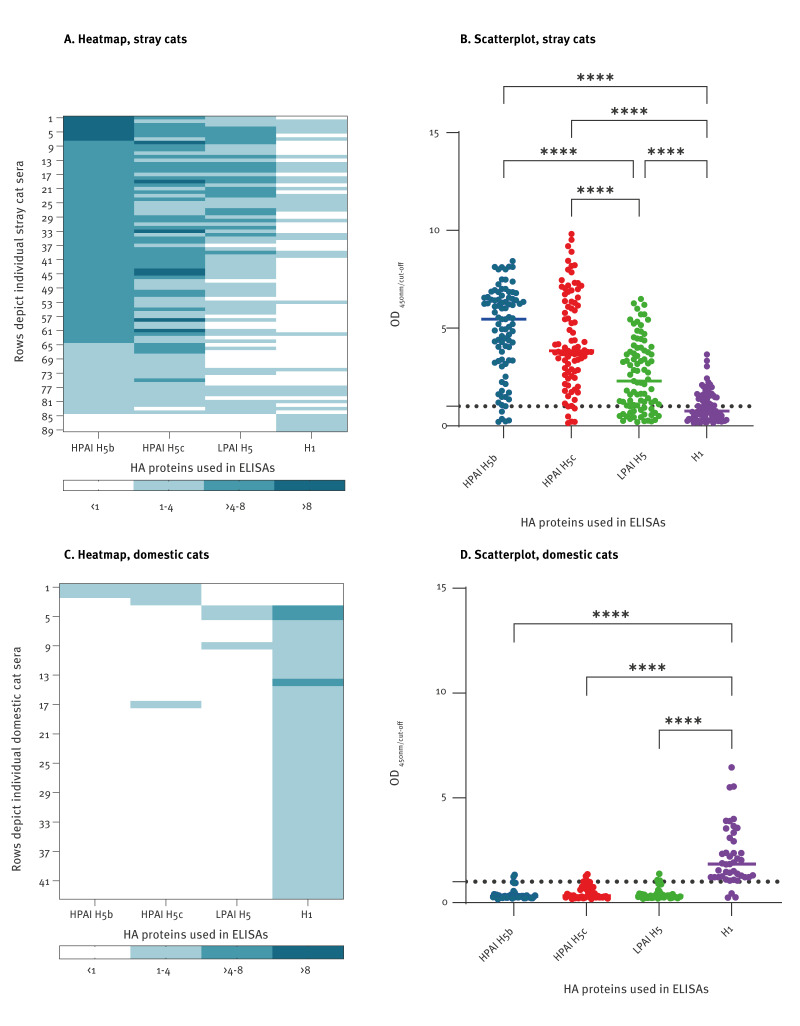
Heat maps and scatterplots presenting serum samples from stray cats (n = 89) and domestic cats (n = 43) tested positive in ELISA for antibodies to highly (HPAI), to low pathogenic avian influenza (LPAI) H5 virus and/or to human influenza H1 virus, the Netherlands, October 2020–June 2023

ELISA: enzyme linked immunosorbent assay; H: hemagglutinin; HA: trimeric haemagglutinin protein of influenza A virus consisting of HA1 and HA2; HPAI: highly pathogenic avian influenza; LPAI: low pathogenic avian influenza; OD: optical density, ELISA OD measured at 450 nm; ****: p value < 0.0001.

All cat sera were analysed in ELISAs. Of the 701 stray cat sera, 89 were ELISA HPAI H5 and/or H1 positive and these were analysed in HAIs. Binding antibodies in the ELISA to HPAI H5c virus were detected in 83 of 701 (11.8%; 95% CI: 9.5–14.5) stray cat sera (Table 1). Of these, 65 of 82 (79.3%) could be confirmed in the HPAI H5 HAI. One serum could not be analysed in HAI due to insufficient volume. All stray cat sera with binding antibodies to HPAI H5c had binding antibodies to HPAI H5b (Figure 2A) with comparable OD ratios (Figure 2B). Additionally, one stray cat serum that reacted borderline to HPAI H5c reacted just above the cut-off for HPAI H5b. Antibodies binding to H1 were present in 35 of 701 (5.0%; 95% CI: 3.5–6.9) stray cat sera (Table 1). The mean rank H1 OD ratio was significantly lower than the mean rank HPAI H5 OD ratios (Figure 2A,B). While 30 of the 35 ELISA H1 seropositive stray cat sera also reacted to HPAI H5c, with higher OD ratios (Figure 2A), five of the 35 stray cat sera exclusively reacted to H1. Only three of these 35 ELISA H1 positive sera were confirmed in the H1 HAI assay, of which two cats were both HAI HPAI H5 and HAI H1 positive. Of the 83 stray cats with binding antibodies to HPAI H5c, 67 (80.7%) reacted in ELISA to LPAI H5, with significantly lower mean rank OD ratios (Figure 2A,B). None of the HPAI H5c and/or H1 positive sera reacted positively in the LPAI H5 HAI, none of the HPAI H5c and H1 negative control sera (0/55) reacted in the HPAI H5b ELISA, LPAI H5 ELISA or in the HAI assays.

**Table 1:** Detection of antibodies against highly pathogenic avian influenza (HPAI) H5 virus and human H1 virus in sera from domestic cats (n = 871) and stray cats (n = 701), the Netherlands, October 2020–June 2023

ELISA antigen	Domestic 1 Nov 2020–Feb 2021		Domestic 2 Jul 2021–Nov 2022		Domestic 3 Sep 2022–Mar 2023		Stray Oct 2020–Jun 2023	
	n	%	n	%	n	%	n	%
HPAI H5c								
Positive	2	0.68	0	0.0	2	0.54	83	11.8
Negative	294	99.3	208	100.0	365	99.5	618	88.2
H1								
Positive	21	7.1	2	1.0	17	4.6	35	5.0
Negative	275	92.9	206	99.0	350	95.4	666	95.0

ELISA: enzyme linked immunosorbent assay; H: hemagglutinin; HPAI: highly pathogenic avian influenza.

In the ELISA tests, HA protein of human H1N1 (H1/California/04/2009) referred to as H1 and HPAI H5N8 clade 2.3.4.4c (A/Chicken/NL/14015526/2014) referred to as HPAI H5.

In the ELISAs, HA proteins of human H1N1pdm2009, avian HPAI H5N8 clade 2.3.4.4c 2014, avian HPAI H5N1 clade 2.3.4.4b 2022 and avian LPAI H5N2 2017 were used. The ELISA cut-off (Five times the OD value of a negative specific pathogen free cat serum) was calculated per assay and the results are depicted as OD ratios (OD value/cut-off).

Panel A and C. Each row represents the reactivity of a single cat serum. The blue colour corresponds with ELISA binding activity of the cat sera based on the OD ratio. The white colour depicts reactivity below the cut-off.

Panel B and D. The dotted line represents the cut-off for ELISA positive samples (OD ratio = 1). The horizontal lines in the scatterplots depict the median. The mean rank OD ratios in the scatterplots were compared using the Kruskal-Wallis test with Dunn’s correction for multiple comparisons.

#### Domestic cats

Of the 871 domestic cat sera, 43 sera positive in ELISA for HPAI H5 and/or H1 were analysed in HAIs. In domestic cats, four of 871 (0.46%; 95% CI: 0.13–1.2) sera had ELISA HPAI H5c-binding antibodies (Table 1) but none could be confirmed in the HPAI H5 HAI (Figure 2C). Binding antibodies to H1 were present in 40 of 871 (4.6%; 95% CI: 3.3–6.2) sera (Table 1), and 26 of 40 were confirmed in the H1 HAI. None of the domestic cat sera reacted positive in the LPAI H5 HAI. The mean rank H1 OD ratio of the domestic cat sera was significantly higher than the mean rank HPAI H5 and LPAI H5 OD ratios (Figure 2D). The number of ELISA H1 positive sera in Domestic 2 was significantly (p = 0.005) lower compared with the other two cohorts. When the sera from Domestic 2 were excluded from the analysis, the ELISA H1 seropositivity in the domestic cats, based on 38 of 663 sera, was 5.7%.

Cross-reactivity among the four HA proteins in the ELISA was investigated using four reference sera, and results are presented in Supplementary Figure S1A, B, C and D. Based on these reference sera, we conclude that in the ELISAs cross-reactivity in cat sera may occur among the H5 proteins, and to a low extent to H1.

### Detection of influenza A virus (IAV)

No IAV was detected by RT-qPCR in the pharyngeal swabs or lung tissue samples of 208 domestic cats or in 22 stray cats with acute respiratory or neurologic clinical signs.

### Analysis of factors associated with exposure to influenza virus

At 20 of the 72 sampling sites, at least one stray cat with HPAI H5c-binding antibodies was found. At five of these sites, three or more cats had HPAI H5c-binding antibodies. These sampling sites were either nature reserves or dairy farms close to nature reserves. The lowest number of seropositive cats at these five sites was three of 12, and the highest number was 20 of 24.

The results of the univariable analysis are presented in Supplementary Table S3. The HPAI H5 seroprevalence was highest in stray cats living on dairy farms (11.0%) and in nature reserves (37.8%) (Table 2). Multivariable analysis showed that stray cats in nature reserves were statistically more likely (OR = 5.4; 95% CI: 1.5–20.1) to be ELISA HPAI H5 seropositive compared with the reference category stray cats in countryside residences, which were overcrowded locations with feral cats [27]. Also, HPAI H5 seropositivity was statistically more likely in cats with an estimated age  ≥ 3 years (OR = 3.8; 95% CI: 2.7–7.1) and was borderline statistically more likely in cats with a positive FIV status (OR = 2.6; 95% CI: 1.0–7.1) (Table 2). When cats sampled in 2023 were excluded from the analysis, the HPAI H5 seroprevalence was 10.1% (vs 11.8%). In the subset of stray cat samples collected from 2020 until 2022, the same significant univariable associations with these factors were found.

**Table 2:** Multivariable analysis of factors associated with seropositivity to highly pathogenic avian influenza (HPAI) H5 virus in stray cats, the Netherlands, October 2020–June 2023 (n = 701)^a,b^

Characteristics	Stray cats	HPAI H5 positive		HPAI H5 negative		OR^c^	95% CI	p value
		n	%	n	%			
Total number of cats	664	79	11.9	585	88.1	NA		
Estimated age (years)								
< 3	522	49	9.4	473	90.6	1	NA	
≥ 3	142	30	21.1	112	78.9	3.8	2.1–7.1	< 0.001
Sex								
Female	362	38	10.5	324	89.5	0.96	0.56–1.7	0.887
Male	302	41	13.6	261	86.4	1	NA	
FIV status								
Positive	34	9	26.5	25	73.5	2.6	1.0–7.1	0.048
Negative	630	70	11.1	560	88.9	1	NA	
Location type								
Countryside residence	73	4	5.5	69	94.5	1	NA	
Industrial area	35	3	8.6	32	91.4	0.86	0.16–4.7	0.86
Holiday park or camp site	130	3	2.3	127	97.7	0.25	0.05–1.3	0.094
Dairy farm	344	38	11.0	306	89.0	1.4	0.44–4.4	0.57
Nature reserve	82	31	37.8	51	62.2	5.4	1.5–20.1	0.011
Sampling year								
2020	86	10	11.6	76	88.4	1	NA	
2021	243	5	2.1	238	97.9	0.18	0.055–0.61	0.006
2022	277	46	16.6	231	83.4	1.6	0.67–3.9	0.29
2023	58	18	31.0	40	69.0	1.2	0.36–4.0	0.77

CI: confidence interval; ELISA: enzyme linked immunosorbent assay; FIV: feline immunodeficiency virus; H: hemagglutinin; HPAI: highly pathogenic avian influenza; NA: not applicable; OR: odds ratio.

^a^ ELISA using the HA protein of A/Chicken/NL/14015526/2014.

^b^ Metadata were available from 664 stray cats.

^c^ Odds ratio was determined by binary logistic regression; constant included in the model.

In the univariable analysis of domestic cats, age (p = 0.172), sex (p = 0.896) and sampling year (p = 0.134) were not significantly associated with the presence of H1-binding antibodies.

## Discussion

In this study, stray cat and domestic cat sera collected in the Netherlands 2020–2023 were analysed for antibodies to HPAI H5 and H1 virus. Our results showed that HPAI H5 exposure was common in stray cats and rare in domestic cats.

The different proportion of seropositivity for HPAI H5 virus in stray cats and domestic cats in our study, may reflect differences in exposure to birds. Given their reliance on predation or scavenging for survival, stray cats that feed on prey, including birds [32], face an increased risk of HPAI H5Nx exposure compared with domestic cats that are fed and predominantly stay indoors.

The HPAI H5 seropositivity of 11.8% in clinically healthy stray cats contrasts with recent case reports describing HPAI H5Nx infections in cats resulting in severe disease and death [8,10,12-15]. However, antibodies to HPAI H5 have been reported in clinically healthy cats [11,18,19,33]. Important explanatory factors for these conflicting results may be related to differences in transmission route, viral dose, or other, not yet known, predisposing factors. Experimental intranasal, oral or enteral infections all resulted in respiratory and extra-respiratory clinical signs, and these cats died or were euthanised within 7 days [20,21,34-36]. However, the occurrence and severity of clinical signs in cats infected with HPAI H5N1 clade 2.2.2 appeared dose-dependent [37]. We therefore hypothesise that the presence of HPAI H5 antibodies in clinically unaffected stray cats or stray cats that survived infection, may be the result of exposure to a low viral dose.

The higher HPAI H5 and lower LPAI H5 ELISA reactivity based on OD ratios in the stray cat sera suggests that the cats were exposed to HPAI H5 rather than to LPAI H5 but cross-reacted in the ELISA tests [38]. This was substantiated by the high proportion (65/82) of HPAI H5 ELISA positive stray cat sera that were HAI confirmed for HPAI H5 while none were confirmed for LPAI H5. The HAI is the gold standard for influenza A virus antibody detection, cross-reactivity is not expected in the HAI assays. We consider the ELISA and HAI as complementary serological assays for the serosurveillance of influenza A virus infections in cats. The HA-ELISA is more sensitive than the HAI, and the HAI is more specific [19]. Like stray cats, wild carnivores had antibodies to HPAI H5 and not to LPAI H5 in the Netherlands between 2020 and 2022 [39]. Furthermore, stray cats may more easily prey on sick or deceased birds infected with HPAI H5 than on clinically unaffected birds infected with LPAI H5. Moreover, the Global Initiative on Sharing All Influenza Data (GISAID, https://gisaid.org) sequence data (2013–2023) on H5 isolates from the Netherlands contain 960 of 996 (96.4%) HPAI H5Nx strains and 36 LPAI H5Nx strains, suggesting a higher circulation of HPAI H5 than LPAI H5 viruses. However, sequencing of these strains could be affected by sampling bias towards HPAI H5 viruses. Although the H1 ELISA seroresponse of domestic cats and stray cats appeared similar in our study, 26 of the 40 serum samples from domestic cats positive for H1 in ELISA were confirmed in the H1 HAI, while only three of the 35 serum samples from stray cats were confirmed. As most ELISA H1 positive stray cat sera were also, and stronger, reactive to HPAI H5, cross-reactivity of HPAI H5 antibodies to the HA protein of H1 may explain the ELISA H1 seroresponse in these cats. Both IAV H5 and IAV H1 belong to IAV group one and are genetically relatively closely related, and as a result there is a substantial amino acid homology in the stem structure [40] to which cross-reactive antibodies are likely to bind [41]. We cannot exclude, however, stray cat exposure to LPAI H1 viruses [38].

The percentage of H1-binding antibodies in domestic cats (4.6%) was lower than in domestic cats sampled in the Netherlands in 2019 (20/131 cats, 15.3%) [19]. This may partly be explained by the low percentage in Domestic 2, where diluted blood clots instead of serum were used. The lower H1 seropositivity may furthermore be explained by a reduced exposure to H1N1pdm2009 from humans due to COVID-19 restrictive measures,  reducing H1N1pdm2009 infections in humans,  2020–2022 [42].

No HPAI H5Nx virus was detected in pharyngeal swabs and lung tissues of domestic cats and stray cats. However, the presence of HPAI H5 in brain tissue or rectum swabs without detection in lung or pharynx, a rare case in wild carnivores in the Netherlands [9], would have remained undetected in our study. Pooling of samples may have resulted in missing positive samples with a very low viral load (Cq > 37). More importantly, based on experimental and natural clinical and subclinical infections in cats, we expect that HPAI H5 virus may only be detected in pharyngeal swabs within 14 days post infection [10,14,15,18,20,21,37].

We found several factors that were associated with an HPAI H5 seropositivity in stray cats. The highest HPAI H5 seropositivity was found in cats living in nature reserves (37.8%) and on dairy farms (11.0%). The higher HPAI H5 seropositivity in stray cats in nature reserves, compared with cats at other locations may be partly explained by the availability of food. Cats in nature reserves depend on scavenging and hunting to survive. Moreover, diseased or dead birds may be removed at locations with human activity, while they may remain longer present in nature. Although avian-to-cat contact may have resulted in HPAI H5 infection in cats living on dairy farms, recent reports point to an alternative route of exposure [8]. In the US, cats on dairy farms with cows infected with HPAI H5N1 became infected via ingestion of raw milk [8]. Cattle infected with HPAI H5 virus have not been detected in the Netherlands, but this needs further investigation. In older cats, a prolonged exposure period or multiple exposures may explain the higher proportion of HPAI H5 seropositivity. Additionally, older cats are more experienced hunters and have larger home ranges [27] which may affect exposure to birds or viruses. Furthermore, antibodies to FIV were detected in 34 of 664 (5.1%; 95% CI: 3.6–7.1%) stray cats. Immunodeficiency due to FIV, reported in stray cats in the Netherlands previously [27], is associated with an enhanced susceptibility to other pathogens. This may explain the higher HPAI H5 seroprevalence in FIV-positive stray cats.

The difference in stray cat HPAI H5 seropositivity needs to be interpreted with caution. The convenience sampling of stray cats during trap-neuter-return-and-care activities and the targeted sampling in 2023 in our study may have resulted in a selection bias [27]. The distribution of sampling locations, sampling month, as well as the location type differed per year. Sampling intact, younger stray cats may have resulted in an underestimation of the HPAI H5 seroprevalence, as older age was associated with an elevated HPAI H5 seroprevalence. Seroprevalence studies on domestic cats that have outdoor access, especially in rural areas, are needed to further assess their risk of HPAI H5 virus exposure.

## Conclusion

Stray cats in the Netherlands are commonly exposed to HPAI H5 with a high seropositivity in cats in nature reserves. We recommend close monitoring of IAV infections in stray cats and in domestic cats that have outdoor access, especially in areas where HPAI H5 virus positive wild birds are present. When these cats present with neurologic or respiratory clinical signs, we advise to handle these cats using personal protective equipment. Further studies should focus on virus excretion to explore if these cats can transmit viruses to other mammals or to humans. Furthermore, our findings warrant further investigation in the potential role of cats as IAV mixing vessels.

## Supplementary Data

356000 Supplementary Material
