# Supplementary material for: Highly pathogenic avian influenza (HPAI) H5 virus exposure in domestic cats and rural stray cats, the Netherlands, October 2020 to June 2023
Source: Euro Surveill. 2024 Oct 31;29(44):2400326. doi: 10.2807/1560-7917.ES.2024.29.44.2400326 (PMC11528901; doi:10.2807/1560-7917.ES.2024.29.44.2400326)
Supplement: Supplementary Material [file 24-00326_DUIJVESTIJN_Supplement.pdf]

Supplementary material

This supplementary material is hosted by *Eurosurveillance* as supporting information alongside the article "**Highly pathogenic avian influenza (HPAI) H5 virus exposure in domestic cats and rural stray cats, the Netherlands, October 2020 to June 2023"** on behalf of the authors, who remain responsible for the accuracy and appropriateness of the content. The same standards for ethics, copyright, attributions, and permissions as for the article apply. Supplements are not edited by *Eurosurveillance,* and the journal is not responsible for the maintenance of any links or email addresses provided therein.

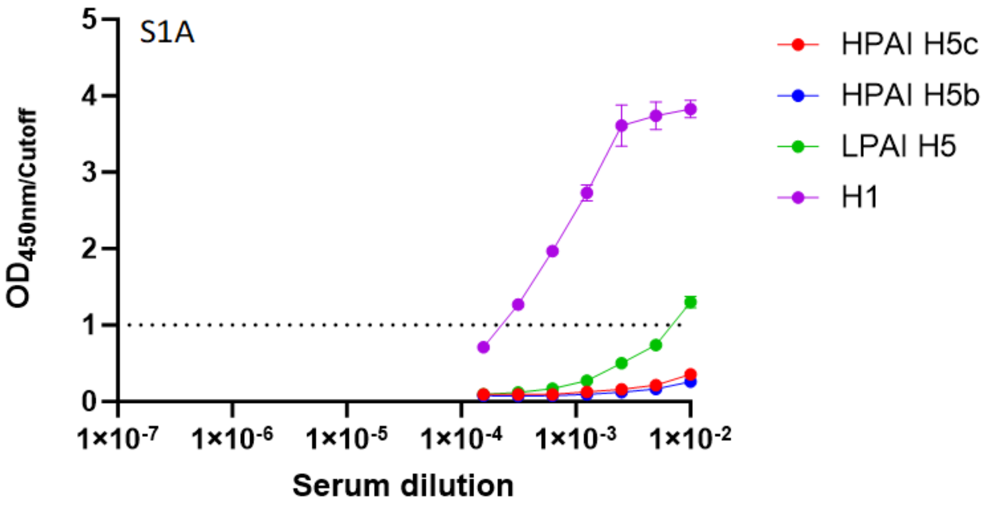

Supplementary Figure S1A. ELISA reactivity of sheep reference antiserum anti-H1N1$_{pdm2009}$ influenza A virus on four HA proteins.

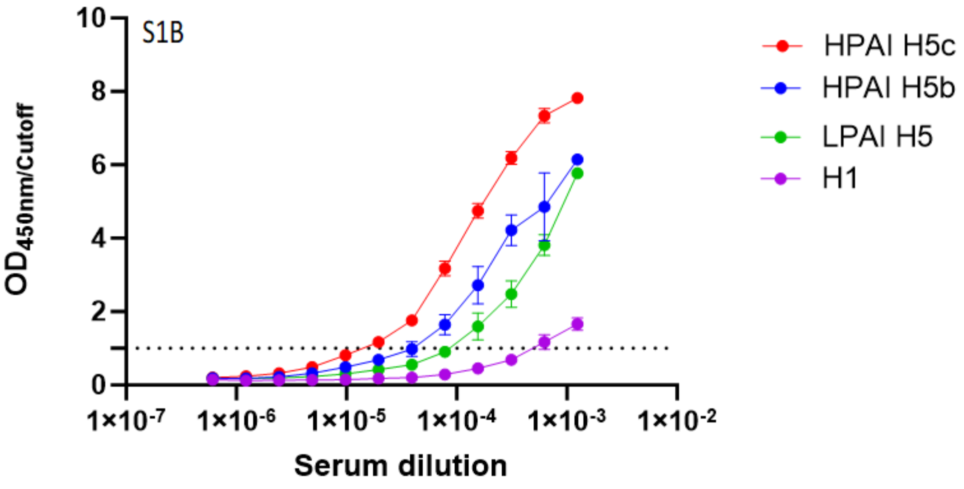

Supplementary Figure S1B. ELISA reactivity of ferret reference antiserum anti-H5N8 influenza A virus clade 2.3.4.4c on four HA proteins.

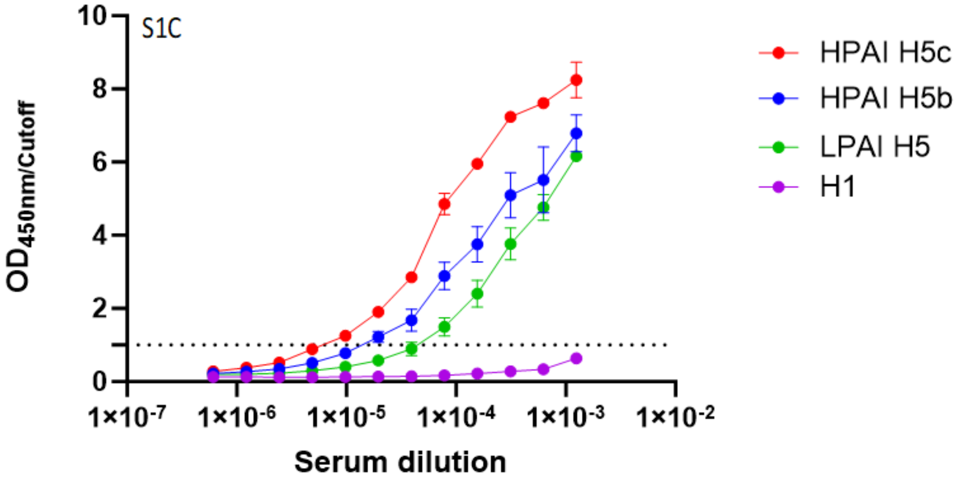

Supplementary Figure S1C. ELISA reactivity of ferret reference antiserum anti-H5N1 influenza A virus clade 2.3.4.4b on four HA proteins.

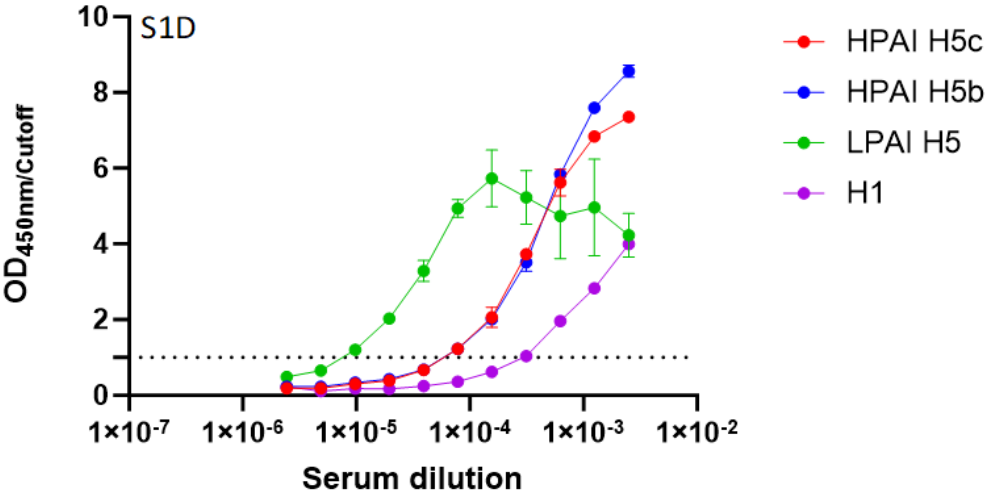

Supplementary Figure S1D. ELISA reactivity of ferret reference antiserum anti-LPAI H5 influenza A virus on four HA proteins.

ELISA: enzyme linked immunosorbent assay. OD: ELISA Optical Density measured at 450 nm. HA: trimeric hemagglutinin protein of influenza A virus; HA1 and HA2. HA proteins of human H1N1pdm2009, avian HPAI H5N8 clade 2.3.4.4c, avian HPAI H5N1 clade 2.3.4.4b and avian LPAI AI H5N2 were used. A description of the IAV subtypes used can be found in Supplementary Table S1. The ELISA cutoff (5* the OD value of a negative SPF cat serum) was calculated per assay and the results are depicted as OD ratios (OD value/cutoff). The dotted line represents the cutoff for ELISA positive samples (OD ratio=1) S1D: the LPAI H5 OD ratio in the serum dilutions 1:800-1:6400 could not be reliable determined due to precipitation in the wells by high concentrations of binding antibodies.

The sheep anti-H1 antiserum showed no cross-reactive binding to HPAI H5b/H5c and low cross-reactive binding to LPAI H5. The ferret anti-HPAI H5b and ferret anti-HPAI H5c antisera showed strong binding to the HPAI H5b/H5c proteins and to LPAI H5 protein, and weak cross-reactive binding to H1. The ferret anti-LPAI H5 antiserum showed strong reactivity to LPAI H5, moderate reactivity to HPAI H5b/H5c and low reactivity to H1.

		HPAI H5b	Anti-HPAI 5b	HPAI H5c	Anti-HPAI 5c	LPAI H5	Anti-LPAI H5	H1	Anti-H1	
HA1 protein	HPAI H5b		1,00	0,97	0,96	0,89	0,89	0,64	0,63	HA protein
	Anti-HPAI H5b	0,99		0,97	0,96	0,89	0,89	0,63	0,63	
	HPAI H5c	0,96	0,96		0,99	0,88	0,89	0,64	0,64	
	Anti-HPAI H5c	0,97	0,97	0,99		0,88	0,88	0,63	0,63	
	LPAI H5	0,85	0,84	0,84	0,84		0,98	0,66	0,66	
	Anti-LPAI H5	0,86	0,85	0,85	0,85	0,98		0,66	0,66	
	H1	0,53	0,52	0,53	0,53	0,55	0,55		0,99	
	Anti-H1	0,52	0,52	0,53	0,53	0,54	0,55	0,99		

Supplementary Figure S1E. Sequence identity matrix based on the full-length HA or HA1 proteins of four influenza A virus (IAV) strains used in the ELISAs and IAV strains used for the reference sera.

Used antigens: hemagglutinin protein: HA protein of human H1N1pdm2009 (A/California/04/2009) referred to as H1, HPAI H5N8 clade 2.3.4.4c (A/Chicken/NL/14015526/2014) referred to as HPAI H5c, HPAI H5N1 clade 2.3.4.4b (A/Common Tern/NL/26/2022) referred to as HPAI H5b and LPAI AI H5N2 (A/Common Teal/NL/4/2022) referred to as LPAI H5. Used antisera: sheep polyclonal reference antiserum H1N1pdm2009 (A/California/7/09 referred to as anti-H1, ferret polyclonal reference antiserum HPAI H5N8 clade 2.3.4.4c (A/Chicken/NL/EMC-3C/2014 referred to as ferret anti-H5c, ferret polyclonal reference antiserum HPAI H5N1 clade 2.3.4.4b (A/European polecat/NL/1/2022 referred to as anti-H5b and ferret polyclonal reference antiserum LPAI AI H5N9 (A/Mallard/Sweden/49/2002 referred to as anti-LP H5. This matrix was generated with BioEdit (version 7.0.9.0).

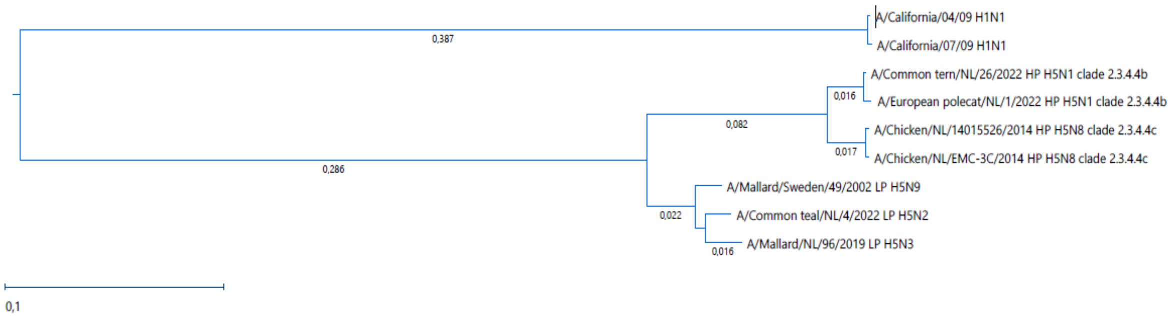

Supplementary Figure S1F: Phylogenetic tree based on the full-length HA proteins of four influenza A virus (IAV) strains used in the ELISAs, and IAV strains used for the reference antisera.

Used antigens: hemagglutinin protein (HA): HA protein of human H1N1pdm2009 (A/California/04/2009) (H1), HPAI H5N8) clade 2.3.4.4c (A/Chicken/NL/14015526/2014) (HPAI H5c), HPAI H5N1 clade 2.3.4.4b (A/COTE/NL/26/2022) (HPAI H5b), LPAI H5N2 (A/Common Teal/NL/4/2022) (LPAI H5). Used antisera: sheep polyclonal reference antiserum H1N1pdm2009 (A/California/7/09) (anti-H1), ferret polyclonal reference antiserum HPAI H5N8 clade 2.3.4.4c (A/Chicken/NL/EMC-3C/2014) (anti-HPAI H5c), ferret polyclonal reference antiserum HPAI H5N1 clade 2.3.4.4b (A/European polecat/NL/1/2022) (anti-HPAI H5b) and ferret polyclonal reference antiserum LPAI H5N9 (A/Mallard/Sweden/49/2002) (anti-LPAI H5). Gisaid/Genbank isolate numbers are described in Supplementary Table S1. This maximum likelihood phylogenetic tree was inferred using central rooting, with bootstrap support values using 1000 replicates (MegAlign Pro7).

Supplementary Table S1. Information on influenza A virus subtypes used for ELISAs and HAIs, and IAV subtypes used for generating reference antisera in this study.

Gisaid/ Genbank number	IAV-subtype	Country	Date	Isolate name	Submitting laboratory/ Organisation	Authors
Genbank ACS45035.1	H1N1pdm	USA	2009	A/California/04/2009	Not specified	Ye,Z. and Zoueva,O.

Genbank FJ969540.1	H1N1pdm	USA	2009	A/California/7/09	Novel Swine-Origin Influenza A (H1N1) Investigation Team	Not specified
GISAID EPI_ISL_15069401	HP H5N1	Netherlands	2022	A/Common Tern/NL/26/2022	Erasmus Medical Center	Not specified
GISAID EPI_ISL_13201074	HP H5N1	Netherlands	2022	A/European Polecat/NL/1/2022	Erasmus Medical Center	Not specified
GISAID EPI_ISL_167905	HP H5N8	Netherlands	2014	A/Chicken/NL/14015526/2014	Wageningen Bioveterinary Research	Heutink, Rene; Harders, Frank; Verschuren-Pritz, Sylvia; Bossers, Alex; Koch, Guus; Bouwstra, Ruth
GenBank KR233690.1	HP H5N8	Netherlands	2014	A/Chicken/NL/EMC-3C/2014	Erasmus Medical Center	Verhagen,J. H., Van der Jeugd,H.P., Nolet,B.A., Vuong,O., Majoor,F., De Vries,P.P., Kharitonov,S., Kuiken,T. and Fouchier,R.A.M.
GenBank GU052868.1	LP H5N9	Netherlands	2002	A/Mallard/Sweden/49/2002	USDA	Kim,L.M., Scott,M.A., Suarez,D.L., Spackman,E., Swayne,D.E. and Afonso,C.L.
GISAID EPI_ISL_15925877	LP H5N2	Netherlands	2022	A/Common_Teal/NL/4/2022	Erasmus Medical Center	Not specified
GISAID EPI_ISL_18537218	LP H5N3	Netherlands	2019	A/Mallard/Netherlands/96/2019	Erasmus Medical Center	Not specified

Supplementary Table S2: Description of primers and probes used in the qPCR for the detection of influenza A virus. Primers and probe used for influenza A virus detection were derived from [1].

Target	Primer/Probe	Sequence 5' - 3'
**Influenza A virus**	IAV-F	AAGACCAATYCTGTCACCTYTGA
	IAV-R	CAAAGCGTCTACGCTGCAGTCC
	IAV-P	TTTGTKTTCACGCTCACCGTGCC
**Phocine distemper virus**	PDV-F	CGG GTG CCT TTT ACA AGA AC
	PDV-R	TTC TTT CCT CAA CCT CGT CC
	PDV-P	Cy5-ATG CAA GGG CCA ATT CTT CCA AGT T-BBQ
**Feline glyceraldehyde-3 phosphate dehydrogenase**	GAPDH-F	GCCGTGVGAATTTGCCGT
	GAPDH-R	GCCATCAATGACCCCTTCAT
	GAPDH-P	CTCAATACATGGTCTACTGTTCCAGTATGATTCCA

Supplementary Table S3: Strength of association, using univariable analysis, of putative risk factors with rural stray cat ELISA HPAI H5 seropositivity in the Netherlands, sampled from 2020 until 2023 (n =701).

Variable (sample size[#])		Total[#]		HPAI H5 pos		HPAI H5 neg		Relative Risk	95% CI[*]	*p*-Value
		Nr.	%	Nr.	%	Nr.	%			
	**Total**	701	100	83	11.8	618	88.2	NA	NA	NA
**Estimated age (n=692)**	**<3 years**	548	79.2	50	9.1	498	90.9	Ref.	NA	NA
	**≥ 3 years**	144	20.8	30	20.8	114	79.2	2.3	1.5-3.5	<0.001
**Sex (n=699)**	**Female**	385	55.1	41	10.6	344	89.4	Ref.		
	**Male**	314	44.9	42	13.4	272	86.6	1.3	0.84-1.9	0.27
**FIV status (n=701)**	**FIV pos**	36	5.2	9	25.0	27	75.0	2.3	1.2-4.2	0.028
	**FIV neg**	663	94.8	73	11.0	590	89.0	Ref.	NA	NA
**Location type (n=675)**	**Dairy farm**	347	5.4	38	11.0	309	89.0	Ref	NA	NA
	**Industrial area**	35	5.2	3	8.6	32	91.4	0.78	0.25-2.4	0.67
	**Countryside residence**	74	11.0	4	5.4	70	94.6	0.49	0.18-1.3	0.17
	**Holiday parc or camp site**	132	19.6	3	2.3	129	97.7	0.21	0.065-0.66	0.0078
	**Nature reserve**	87	12.9	35	40.2	52	59.8	3.7	2.5-5.5	<0.001
**Sampling year (n=701)**	**2020**	87	12.4	10	11.5	77	88.5	0.66	0.35-1.2	0.20
	**2021**	267	38.1	5	1.9	262	98.1	0.11	0.044-0.27	<0.001
	**2022**	288	41.1	50	17.4	238	82.6	Ref		
	**2023**	59	8.4	18	30.5	41	69.5	1.8	1.1-2.8	0.016

CI: confidence interval. ELISA: enzyme linked immunosorbent assay. FIV: feline immunodeficiency virus specific antibodies, detected using commercial antibody ELISA. HPAI

H5: Influenza A virus H5 clade 2.3.4.4c antibodies detected using ELISA. NA: not applicable. Pos: positive. Neg: negative. Ref: reference category. Nr: number. *95% CI for the Relative Risk. #Not all serum samples were accompanied by all metadata. Therefore, the total numbers within a variable can be lower than n=701.

References

[1] Munster VJ, Baas C, Lexmond P, Bestebroer TM, Guldemeester J, Beyer WEP, et al. Practical Considerations for High-Throughput Influenza A Virus Surveillance Studies of Wild Birds by Use of Molecular Diagnostic Tests. Journal of Clinical Microbiology 2009. https://doi.org/10.1128/jcm.01625-08.
